# An immune-related gene signature predicts the 28-day mortality in patients with sepsis

**DOI:** 10.3389/fimmu.2023.1152117

**Published:** 2023-03-23

**Authors:** Yaojun Peng, Qiyan Wu, Hongyu Liu, Jinying Zhang, Qingru Han, Fan Yin, Lingxiong Wang, Qi Chen, Fei Zhang, Cong Feng, Haiyan Zhu

**Affiliations:** ^1^ Department of Graduate Administration, Medical School of Chinese People's Liberation Army (PLA) General Hospital, Beijing, China; ^2^ Department of Emergency, The First Medical Center, Chinese People's Liberation Army (PLA) General Hospital, Beijing, China; ^3^ Institute of Oncology, The Fifth Medical Centre, Chinese People's Liberation Army (PLA) General Hospital, Beijing, China; ^4^ Department of Neurosurgery, The First Medical Center, Chinese People's Liberation Army (PLA) General Hospital, Beijing, China; ^5^ Department of Neurosurgery, Hainan Hospital of Chinese People's Liberation Army (PLA) General Hospital, Sanya, Hainan, China; ^6^ Department of Basic Medicine, Medical School of Chinese People's Liberation Army (PLA) General Hospital, Beijing, China; ^7^ Department of Oncology, The Second Medical Center & National Clinical Research Center of Geriatric Disease, Chinese People's Liberation Army (PLA) General Hospital, Beijing, China; ^8^ Department of Traditional Chinese Medicine, The First Medical Center, Chinese People's Liberation Army (PLA) General Hospital, Beijing, China

**Keywords:** sepsis, prognosis, immune-related genes, transcriptomic profile, integrative analysis

## Abstract

**Introduction:**

Sepsis is the leading cause of death in intensive care units and is characterized by multiple organ failure, including dysfunction of the immune system. In the present study, we performed an integrative analysis on publicly available datasets to identify immune-related genes (IRGs) that may play vital role in the pathological process of sepsis, based on which a prognostic IRG signature for 28-day mortality prediction in patients with sepsis was developed and validated.

**Methods:**

Weighted gene co-expression network analysis (WGCNA), Cox regression analysis and least absolute shrinkage and selection operator (LASSO) estimation were used to identify functional IRGs and construct a model for predicting the 28-day mortality. The prognostic value of the model was validated in internal and external sepsis datasets. The correlations of the IRG signature with immunological characteristics, including immune cell infiltration and cytokine expression, were explored. We finally validated the expression of the three IRG signature genes in blood samples from 12 sepsis patients and 12 healthy controls using qPCR.

**Results:**

We established a prognostic IRG signature comprising three gene members (*LTB4R*, *HLA-DMB* and *IL4R*). The IRG signature demonstrated good predictive performance for 28-day mortality on the internal and external validation datasets. The immune infiltration and cytokine analyses revealed that the IRG signature was significantly associated with multiple immune cells and cytokines. The molecular pathway analysis uncovered ontology enrichment in myeloid cell differentiation and iron ion homeostasis, providing clues regarding the underlying biological mechanisms of the IRG signature. Finally, qPCR detection verified the differential expression of the three IRG signature genes in blood samples from 12 sepsis patients and 12 healthy controls.

**Discussion:**

This study presents an innovative IRG signature for 28-day mortality prediction in sepsis patients, which may be used to facilitate stratification of risky sepsis patients and evaluate patients’ immune state.

## Introduction

Sepsis affects approximately 49 million people each year, and an estimated 11 million people die of sepsis, accounting for 19.7% of all global deaths (1). Sepsis is defined as a heterogeneous clinical condition with complicated immune pathophysiology where a “genomic storm” that dynamically alters the leukocyte transcriptome is provoked by an invading pathogen, with the activation of the innate immune response and a concomitant suppression of the adaptive immune response (2). The early transcriptional changes occur within hours of sepsis initiation and are reportedly predictive of later clinical outcomes of the hosts (3). However, a comprehensive characterization of the immune response in sepsis has not been fully uncovered, and its clinical relevance deserves further exploration.

The early and accurate diagnosis and risk stratification of sepsis remains a challenge. Procalcitonin, for example, has shown clinical utility in guiding antibiotic usage in the setting of lower respiratory tract infections (4), but its predictive and prognostic capability in sepsis is controversial (5, 6). Several investigators have successfully utilized leukocyte-derived mRNA and bioinformatics approaches to subgroup sepsis patients on the basis of biological similarities defined by transcriptomic signature (7). Transcriptomics signature, as one of the promising new biomarkers, is seemingly able to aid more for predictive and prognostic purposes (8).

In the current study, by analyzing the transcriptomic data of peripheral blood mononuclear cells (PBMCs) from sepsis patients, we identified functional immune-related genes (IRGs) and constructed a model for predicting the 28-day mortality of sepsis patients, namely the IRG signature. The prognostic value of the model was validated in internal and external sepsis datasets. The correlations of the IRG signature with immunological characteristics, including immune cell infiltration and cytokine expression, were explored. We also performed pathway analysis to uncover the underlying biological mechanisms of the IRG signature. Finally, experimental verification of the IRG signature genes was carried out in blood samples from sepsis patients and healthy controls.

## Methods

### Data acquisition and preprocessing

The gene expression profiles of whole-blood leukocytes from sepsis patients and the corresponding clinical information contained in GSE65682 (9) and E-MTAB-4451 (10) datasets were downloaded from the Gene Expression Omnibus (GEO, https://www.ncbi.nlm.nih.gov/geo/) and the ArrayExpress (https://www.ebi.ac.uk/arrayexpress/) databases, respectively. In GSE65682, blood samples were taken within 24 h of admission to critical care and subjected to the GPL570 Affymetrix Human Genome U133 Plus 2.0 Array (HGU133_Plus_2) platform for gene-expression quantification. A total of 479 sepsis patients with available 28-day follow-up information and 42 healthy controls in GSE65682 were included. In E-MTAB-4451, blood samples of sepsis patients were obtained after ICU admission at the time of study enrolment and sequenced on the GPL10558 Illumina HumanHT-12 V4.0 expression beadchip platform. Totally, 106 cases of sepsis patients with available 28-day follow-up information in E-MTAB-4451 were included. The baseline characteristics of sepsis patients included in this study are summarized in **Table 1**. For gene expression data preprocessing, probes were transformed into gene symbols based on the annotation file provided by the platform manufacturer. In particular, probes without corresponding gene symbols were removed, and average values were obtained if one gene corresponded to multiple probes.

**Table 1:** Baseline characteristics of sepsis patients included in this study.

GSE65682 (n = 479)	
Age, years	60.95 (14.79)
Gender	
Male	272 (56.78%)
Female	207 (43.22%)
DM	
No	301 (62.84%)
Yes	89 (18.58%)
NA	89 (18.58%)
Source of infection	
CAP	106 (22.13%)
HAP	77 (16.08%)
Abdomen	48 (10.02%)
NA	248 (51.77%)
Thrombocytopenia	
Normal	24 (5.01%)
Low	24 (5.01%)
Medium low	30 (6.26%)
Very low	17 (3.55%)
NA	384 (80.17%)
Mars endotype	
Mars1	132 (27.56%)
Mars2	176 (36.74%)
Mars3	118 (24.63%)
Mars4	53 (11.07%)
28-day mortality event	
Alive	365 (76.20%)
Dead	114 (23.80%)
Follow-up time, days	23.19 (9.20)
E-MTAB-4451 (n = 106)	
Age, years	69.08 (14.36)
Gender	
Male	27 (25.47%)
Female	79 (74.53%)
SRS group	
SRS1	37 (34.91%)
SRS2	69 (65.09%)
28-day mortality event	
Alive	54 (50.94%)
Dead	52 (49.06%)

Data are n (%) or mean (SD) unless otherwise specified. DM, Diabetes mellitus; MARS, the Molecular Diagnosis and Risk Stratification of Sepsis; SRS, sepsis response signature; NA, not available.

### Differential expression analysis

Differential expressed genes (DEGs) between sepsis patients and healthy controls were identified using the *limma* R package. Genes with mean expression value of zero were filtered out. The gene matrix was normalized with the *normalizeBetweenArrays* function of the *limma* R package. The screening criteria were log_2_ |fold change (FC)| ≥ 1 and adjusted (adj.) P-value < 0.05.

### Weighted gene co-expression network analysis

Weighted gene co-expression network analysis (WGCNA) is a systematic bioinformatics method which constructs a free-scale network to explore the interrelationship between genes within the corresponding gene module and associations between gene modules and clinical traits (11). In this study, genes with standard deviation of expression level more than 50% were selected for WGCNA analysis. Sample hierarchical clustering was performed followed by establishment of a weighted correlation network using the *WGCNA* R package (12). For the scale-free network, the soft power of R^2^ was set depending on the scale independence and mean connectivity. Genes with similar patterns were clustered into the same modules (minimum size = 50) using average linkage hierarchical clustering, and modules with highly correlated eigengenes were merged with a minimum module merging height of 0.25. Gene significance and module membership were assessed to validate the stability, and correlations between clinical traits and gene modules were evaluated using Pearson’s correlation analysis. The module with the highest positive correlation coefficient was chosen for further analysis.

### Intersection analysis

A list of IRGs was downloaded from the Immunology Database and Analysis Portal (ImmPort, https://www.immport.org) (**Table S1**). The IRGs, DEGs between sepsis patients and healthy controls, and the genes in the most positively correlated WGCNA module were subjected to an intersection analysis to obtain functional IRGs closely related to sepsis by drawing a Venn diagram.

### Pathway enrichment analysis

In order to explore the potential pathways the functional WGCNA module involved, genes of the selected module were extracted and subjected to enrichment analysis using Metascape, a free online tool for gene annotation (http://metascape.org) (13). To explore the potential pathways the IRG signature involved, the DEGs between the IRG signature subgroups were identified for pathway enrichment, which was carried out by Gene Ontology (GO) functional annotation into three major categories: BP (Biological Process), CC (Cellular Component), and MF (Molecular Function). The GO analysis was performed using the *clusterProfiler* R package (14).

### Construction and validation of a prognostic IRG signature

The GSE65682 dataset was used to train and test a prognostic IRG signature, while the E-MTAB-4451 dataset was used to externally validate the predictive capability of the identified IRG signature. The GSE65682 dataset was randomly assigned to the training (n = 240) and testing (n = 239) sets at a 1:1 ratio. Univariate Cox regression analysis was performed in the training set to identify IRGs of prognostic value with P-value < 0.05 as the screening threshold. The LASSO estimation was further applied to penalize the effect of multicollinearity using the *glmnet* R package (15). Subsequently, IRGs reaming from the LASSO estimation were subjected to multivariate Cox regression to construct a best-fitting prognostic model, with the Akaike information criterion (AIC) indicating model fitness and the Harrell’s concordance index (C-index) indicating the discrimination ability (16). Above steps in the multivariate Cox regression analysis were executed by the *survminer* R package. The product of the multivariate Cox regression coefficient *βi* of each gene and the corresponding gene expression *i* were added to establish the risk score formula: $\text{risk score}=\sum_{i=1}^{n}\beta i*i$ .

The risk score of each patient in the GSE65682 and E-MTAB-4451 datasets was calculated according to the risk score formula. Patients were categorized into high risk or low risk subgroups based on the median score of the corresponding dataset. The prognostic performance of the identified IRG signature was internally tested in the testing set (n = 239) and entire set (training set plus testing set, n = 479) of GSE65682, and externally validated in E-MTAB-4451 (n = 106). Survival differences between the low and high risk subgroups were assessed by the Kaplan-Meier estimate. The receiver operating characteristic (ROC) analysis was performed to assess the sensitivity and specificity of the survival prediction based on the risk score using the *survivalROC* R package.

### Estimation of immune cell subtypes

CIBERSORT is a well-established technique for estimating the immune cell compositions of “bulk tissue” from gene-expression data (17). In this study, the relative abundances of 22 subtypes of immune cells in samples were calculated using the *CIBERSORT* R package. The Mann-Whitney U test was used to compare the differences between the IRG subgroups.

### Analysis of cytokines and chemokines

A panel of 27 clinically detectable inflammatory cytokines and chemokines (**Table S2**) was collected from a published study (18). The expression values of these cytokines and chemokines were extracted from the GSE65682 dataset and were further compared between the IRG subgroups. Differences were analyzed using the Mann-Whitney U test. Additionally, Spearman correlation analysis was used to explore the relationship between the IRG signature risk score and the ratio of *IL10*/*TNF*.

### Validation experiments in clinical specimens

The study was reviewed and approved by the Institutional Research Ethics Committee of the PLA General Hospital. A signed informed consent was obtained for each of the participants. Quantitative real-time PCR (qPCR) was used to verify the expression of the three genes that comprised the IRG signature. PBMCs were isolated by density centrifugation of buffy coats from blood samples of 24 clinical specimens, including 12 sepsis samples and 12 healthy controls. Total RNA of PBMCs was extracted by the TRIzol method (19). Reverse transcription of RNA was completed using a RevertAid RT Reverse Transcription Kit (Thermo Scientific, Waltham, MA). qPCR was performed on the StepOnePlus Real-Time PCR System (Applied Biosystems, Foster City, CA) by monitoring the fluorescence of SYBR Green (TaKaRa BioInc, Dalian, China) binding to double-stranded DNA. The settings for the PCR thermal were as follows: initial denaturation at 95°C for 30 s, followed by 40 amplification cycles of 95°C for 5 s, 60°C for 15 s, and 72°C for 15 s. A dissociation analysis was performed at the end of each PCR reaction to ensure its specificity. Each PCR was run in triplicate. For quantification of gene expression changes, the 2^–ΔΔCt^ method was used to calculate relative fold changes normalized against the GAPDH gene. Primers for qPCR are listed in **Table S3**.

### Statistical analysis

Statistical analyses were conducted by R software (version 3.6.3) using the corresponding R package mentioned above or GraphPad Prism (version 9.0.0). The R scripts utilized in this study exist at GitHub (https://github.com/pengyaojun0903/sepsis.git). P values < 0.05 were considered statistically significant. All statistical tests were two sided.

## Results

### Construction of a prognostic IRG signature in sepsis

**Figure 1:**
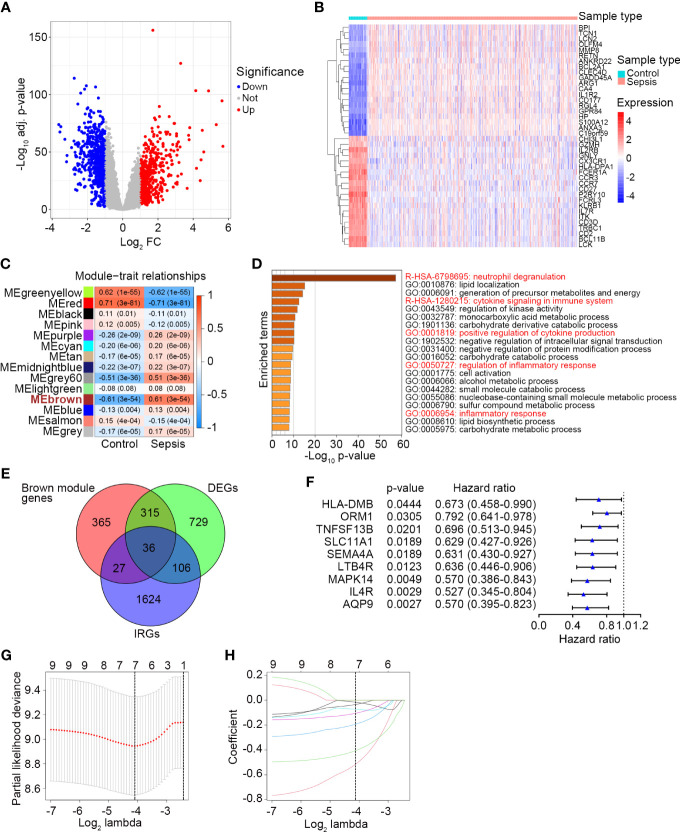
Identification of a prognostic IRG signature in sepsis. **(A)** The volcano plot showing DEGs between healthy controls (n = 42) and sepsis patients (n = 479) in GSE65682. The screening criteria were set as adjusted P-value < 0.05 and |Log_2_ FC| ≥ 1. **(B)** The expression profile of the most significant 20 upregulated and downregulated DEGs. **(C)** Plot of module–trait relationships by WGCNA analysis in the GSE65682 dataset. Each row represents a color module, and each column represents a clinical trait (healthy or sepsis). Each cell contains the corresponding correlation and P-value. ME, module eigengenes. **(D)** Top 20 enriched terms of the genes in the brown module analyzed by Metascape. **(E)** The Venn diagram of intersected genes of brown module genes, DEGs between healthy control and sepsis, and IRGs. **(F)** Univariate Cox regression analysis to screen IRGs related to 28-day survival of sepsis patients in the training set (n = 240) of GSE65682. Blue triangles represent the hazard ratio of death and open-ended horizontal lines represent the 95% confidence intervals. All P-values were calculated using Cox proportional hazards analysis. **(G)** Ten-fold cross-validation for tuning parameter selection in the LASSO estimation. The partial likelihood deviance corresponding to each lambda value is shown as mean ± SD. The dotted vertical line (left) indicates the optimal value by minimum criteria. **(H)** The LASSO coefficient profile of individual genes included in the estimation.

First, we performed differential expression analysis in the GSE65682 dataset to determine DEGs between sepsis patients (n = 479) and healthy controls (n = 42). According to the screening criteria (log_2_ |FC| ≥ 1 and adj. P-value < 0.05), a total of 1185 significant DEGs were obtained with 528 genes upregulated and 657 genes downregulated (**Figure 1A**, **Table S4**) in sepsis patients. The expression profile of the most significantly downregulated and upregulated 20 DEGs is shown in **Figure 1B**. Next, WGCNA was performed to detect functional modules closely related to sepsis (**Figure S1**). A total of 14 modules were detected, and the brown module was the one with the highest positive correlation with sepsis (Pearson r = 0.61, P = 3e-54; **Figure 1C**). There were 743 genes in the brown module (**Table S5**). The enrichment analysis revealed that they were mainly involved in immune-related pathways and metabolic processes (**Figure 1D**). The complete list of the enriched terms is shown in **Table S6**. Since the brown module was implicated in several immune-related biological processes, it was used to inform the selection of IRGs closely related to sepsis. IRGs retrieved from the ImmPort (n = 1793; **Table S1**), DEGs of sepsis (n = 1185; **Table S4**), and genes in the brown module (n = 743; **Table S5**) were applied to the intersection analysis. A total of 36 IRGs were selected (**Figure 1E**), which were further subjected to survival analyses to identify prognostic genes and to fit a risk model. The GSE65682 dataset was randomly assigned to the training (n = 240) and testing (n = 239) sets at a 1:1 ratio. Univariate Cox regression analysis performed in the training set revealed that 9 IRGs were significantly correlated with the 28-day mortality of sepsis patients (**Figure 1F**). 7 of these 9 IRGs were selected through the LASSO method (**Figure 1G, H**), and the subsequent multivariate Cox regression analysis finally determined a prognostic IRG signature consisting of 3 genes (*LTB4R*, *HLA-DMB* and *IL4R*). The full list of genes in the IRG signature is shown in **Table 2**.

**Table 2:** Three gene members of the IRG signature associated with 28-day mortality of sepsis patients.

Gene symbol	Gene name	Chromosome	Coefficient	P-value
*HLA-DMB*	Major histocompatibility complex, class II, DM beta	6p21.32	-0.685	0.002
*IL4R*	Interleukin 4 receptor	16p12.1	-0.668	0.006
*LTB4R*	Leukotriene B4 receptor	14q12	-0.298	0.135

IRG, immune-related gene; AIC, Akaike information criterion; C-index, concordance index; CI, confidence interval.

Global P-value = 9.120e-5; AIC = 542.53, C-index (95% CI) = 0.696 (0.618 to 0.775)

**Figure 2:**
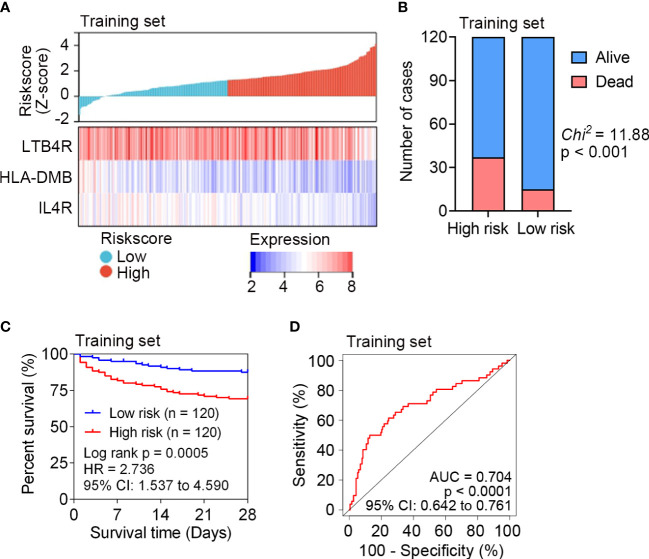
Survival analysis of the IRG signature in the training set (n = 240) of GSE65682. **(A)** The distribution of risk scores derived from the IRG signature, and the expression of the three genes that comprised the IRG signature in the training set of GSE65682. **(B)** The distribution of 28-day survival status in the high risk (n = 120) and low risk (n = 120) subgroups in the training set. Sepsis patients were divided into low risk or high risk subgroup based on the median of risk score. **(C)** Kaplan-Meier estimate of the 28-day survival using the IRG signature in the training set. The difference between the two curves was determined by the two-side log-rank test. **(D)** ROC analysis of the sensitivity and specificity of 28-day mortality prediction by the IRG signature in the training set. P value was obtained from the comparison of the AUC of the IRG signature risk score versus whose classification by 0.5 probability.

The risk score of each patient in the training set (n = 240) was calculated according to the risk score formula. Sepsis patients were divided into a high risk (n = 120) or a low risk (n = 120) subgroup using the median risk score as the cutoff point. Patients with higher risk scores tended to express lower levels of *LTB4R*, *HLA-DMB* and *IL4R*, indicating that upregulation of the three IRGs was a favorable prognostic factor in sepsis (**Figure 2A**). The survival status of sepsis patients in the low risk (n = 120) and high risk (n = 120) subgroups is shown in **Figure 2B**, and higher mortality rate was observed in the high risk subgroup. Kaplan Meier analysis revealed that a significantly inferior 28-day survival was reflected in the high risk subgroup (P = 0.0005, **Figure 2C**). ROC curve was plotted for the training set, and the area under the ROC curve (AUC) was 0.704 (**Figure 2D**).

**Figure 3:**
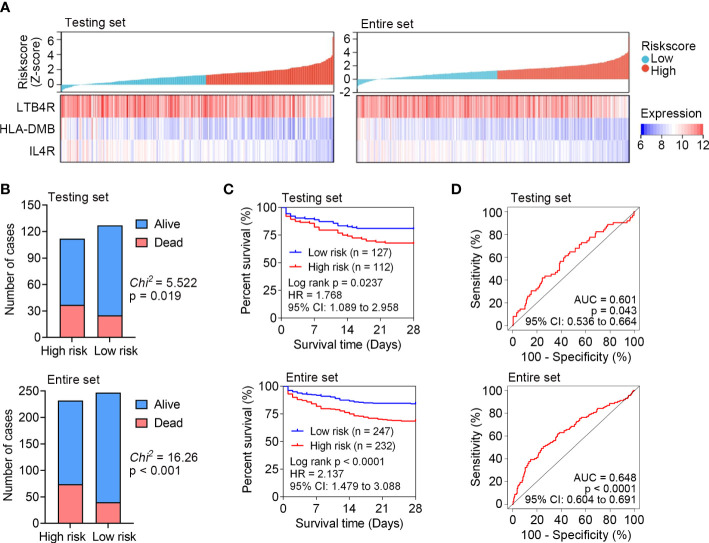
Internal test the predictive capability of IRG signature in the testing set (n = 239) and entire set (n = 479) of GSE65682. **(A)** The distribution of risk scores derived from the IRG signature, and the expression of the three genes that comprised the IRG signature in the testing set and entire set. **(B)** The distribution of 28-day survival status in the high risk and low risk subgroups in the testing set and entire set. **(C)** Kaplan-Meier curves of the 28-day survival based on the IRG signature in the testing set and entire set. The difference between the two curves was determined by the two-side log-rank test. **(D)** ROC analyses of the sensitivity and specificity of 28-day mortality prediction by the IRG signature in the testing set and entire set. P values were obtained from the comparisons of the AUC of the IRG signature risk score versus whose classification by 0.5 probability.

The predictive capability of the identified IRG signature was internally tested in the testing set (n = 239) and entire set (n = 479) of GSE65682. The expression patterns of *LTB4R*, *HLA-DMB* and *IL4R* in the testing set and entire set were the same with that in the training set (**Figure 3A**). The survival status of each patient in the testing set and entire set is shown in **Figure 3B**, and higher mortality rate was observed in the high risk subgroup in both the testing set and entire set. Kaplan Meier analyses revealed significantly inferior 28-day survival in the high risk subgroup in both the testing set and entire set (both P < 0.05; **Figure 3C**). ROC curves were plotted for the testing set and entire set, and AUCs were 0.601 and 0.648, respectively (**Figure 3D**). These results demonstrated that the IRG signature could subdivide sepsis patients into subgroups with significantly disparate survival.

### External validation of the identified IRG signature

**Figure 4:**
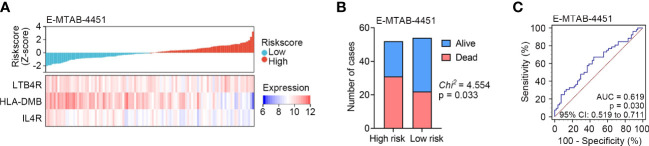
External validation of the identified IRG signature in E-MTAB-4451 (n = 106). **(A)** The distribution of risk scores derived from the IRG signature, and the expression of the three genes that comprised the IRG signature in E-MTAB-4451. **(B)** The distribution of 28-day survival status in the high risk (n = 52) and low risk (n = 54) subgroups in E-MTAB-4451. **(C)** ROC analyses of the sensitivity and specificity of 28-day mortality prediction by the IRG signature in E-MTAB-4451. P value was obtained from the comparison of the AUC of the IRG signature risk score versus whose classification by 0.5 probability.

To further verify the predictive capability of the identified IRG signature, external validation was performed in another sepsis dataset, namely E-MTAB-4451 (n = 106). We calculated the risk score for each patient in the E-MTAB-4451 dataset using the same formula. According to the median risk score, sepsis patients were divided into high risk (n = 52) and low risk (n = 54) subgroups. Lower expression levels of *LTB4R*, *HLA-DMB* and *IL4R* were found in patients with higher risk scores in E-MTAB-4451 (**Figure 4A**), and higher mortality was observed in the high risk subgroup (**Figure 4B**). The AUC of the IRG signature in E-MTAB-4451 was 0.619 (**Figure 4C**). As expected, our validation confirmed that the IRG signature could predict the prognosis of sepsis patients.

### The IRG signature is an independent prognostic factor of sepsis

The relationship between clinical features and the identified IRG signature was analyzed. In GSE65682, four classes of endotype for sepsis, designated as Mars1 - 4, were identified by Scicluna et al. (9) on the basis of whole-blood RNA expression profiles. The Mars endotype was associated with 28-day mortality of sepsis, and the worst outcome was found for patients classified as having a Mars1 endotype (9). In the present study, the IRG signature was significantly associated with source of infection and Mars endotype, but not age, gender, history of diabetes mellitus, or thrombocytopenia (**Table 3**). In E-MTAB-4451, two distinct sepsis response signature groups (SRS1 and SRS2) were defined by Davenport et al. (10) based on transcriptomic analysis of peripheral blood leucocytes, and the presence of SRS1 was associated with higher short-term (14 day and 28 day) and long-term (6 month) mortality than was SRS2. In our study, the IRG signature was significantly associated with gender and SRS group (**Table 3**).

**Table 3:** Correlations between clinical characteristics and the identified IRG signature.

Variable	No.	High risk	Low risk	P value
GSE65682				
Age (years)	479			0.399
≤ 60		98	95	
> 60		134	152	
Gender	479			0.107
Female		109	98	
Male		123	149	
Diabetes mellitus	390			0.548
No		143	158	
Yes		39	50	
Source of infection	231			0.034*
Lung (CAP + HAP)		74	109	
Abdomen		28	20	
Thrombocytopenia	95			0.160
No		10	14	
Yes		42	29	
Endotype	479			< 0.001***
Mars1		102	30	
Mars2		73	103	
Mars3		39	79	
Mars4		18	35	
E-MTAB-4451				
Age (years)	106			0.343
≤ 60		13	9	
> 60		39	45	
Gender	106			0.014*
Female		19	8	
Male		33	46	
SRS group	106			< 0.001***
SRS1		27	10	
SRS2		25	44	

P values were acquired by Chi-square test or Fisher’s exact test. *P < 0.05, ***P < 0.001. CAP, community-acquired pneumonia; HAP, hospital-acquired pneumonia; Mars, molecular diagnosis and risk stratification of sepsis; SRS, sepsis response signature.

**Figure 5:**
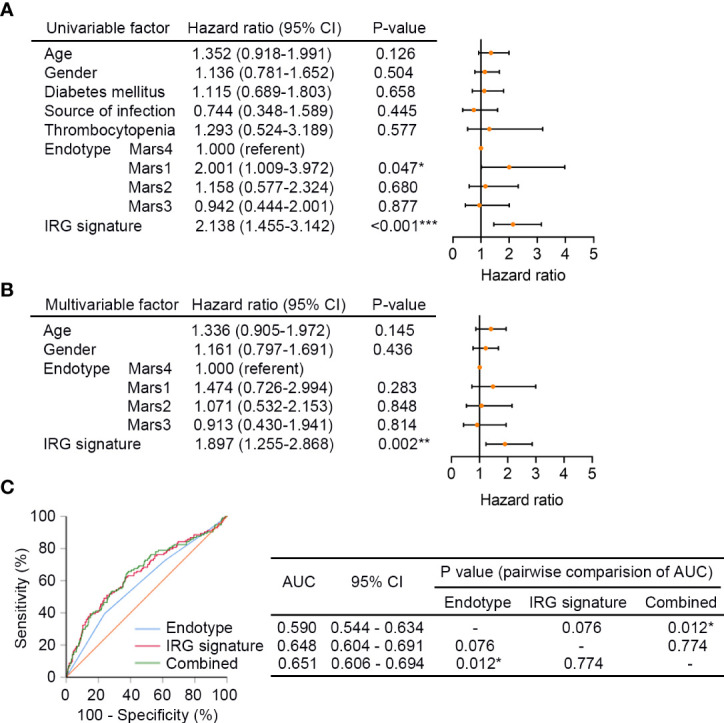
The IRG signature is an independent prognostic factor of sepsis. **(A)** The univariate Cox regression analysis performed on sepsis patients in GSE65682 (n = 479). Orange solid dots represent the hazard ratio of death and open-ended horizontal lines represent the 95% confidence intervals (CIs). All P values were calculated using Cox proportional hazards analysis. **(B)** The multivariate Cox regression analysis performed on sepsis patients in GSE65682 that contained age, gender, Mars endotype, and IRG signature as covariates. **(C)** ROC analysis of the sensitivity and specificity of 28-day survival prediction by the IRG signature risk score, Mars endotype, and combination of the two factors. P values were obtained from the comparisons of the AUC of the Mars endotype versus those of the IRG signature risk score and Mars endotype combined with the IRG signature risk score. *P < 0.05, **P < 0.01, ***P < 0.001. Mars, molecular diagnosis and risk stratification of sepsis.

Next, univariate Cox regression analysis was performed in GSE65682 (n = 479) to screen prognostic clinical characteristics, and the result showed that the Mars endotype (Mar1, P = 0.047, HR = 2.001, 95% CI: 1.009 - 3.972) and the IRG signature (P < 0.001, HR = 2.138, 95% CI: 1.455 - 3.142) were significantly associated with the 28-day survival of sepsis patients (**Figure 5A**). Furthermore, the multivariate Cox regression analysis with age, gender, Mars endotype, and IRG signature included showed that only the IRG signature could serve as an independent prognostic factor of 28-day mortality in sepsis (P = 0.002, HR = 1.897, 95% CI: 1.255 - 2.868; **Figure 5B**). Additionally, we performed ROC analysis to compare the sensitivity and specificity of 28-day mortality prediction between the IRG signature, Mars, and the combination of these two factors. As shown in **Figure 5C**, the AUC of the IRG signature was greater than Mars endotype, though the difference was not statistically significant (0.648 versus 0.590, P = 0.076). Additionally, the AUC of IRG signature combined with Mars endotype was significantly superior to Mars endotype (0.651 versus 0.590, P = 0.012) alone. These results indicated that the IRG signature was an independent prognostic factor of 28-day survival in sepsis, and combination of the IRG signature and Mars endotype may help improve survival prediction in sepsis patients. We also performed ROC analysis in E-MTAB-4451 (n = 106), and the result showed that there was no substantial difference in AUCs of SRS group and IRG signature (0.610 versus 0.619, P = 0.882; **Figure S2**), suggesting the predictive efficacy of IRG signature was equal to that of SRS group identified by Davenport and his colleagues.

### Distribution of immune cell subtypes and expression of cytokines and chemokines in the IRG subgroups

**Figure 6:**
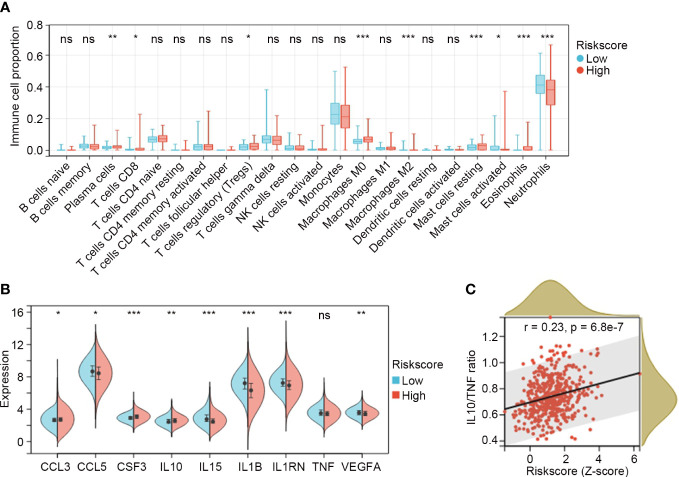
Distribution of immune cell subtypes and expression of cytokines and chemokines in the IRG subgroups. **(A)** Comparisons of immune cell fractions between low risk (n = 247) and high risk (n = 232) IRG subgroups in GSE65682. The Mann-Whitney U test was used to compare the differences. **(B)** Comparisons of cytokines and chemokines between low risk and high risk IRG subgroups. Differences were determined by the Mann-Whitney U test. **(C)** The relation between the IRG risk score and the ratio of *IL10*/*TNF* in sepsis by Spearman correlation analysis. *P < 0.05, **P < 0.01, ***P < 0.001, ns, no significance.

CIBERSORT was applied to GSE65682 to explore the proportions of immune cells in sepsis. The overall distribution of cell fractions is illustrated in **Figure S3**. In the high risk subgroup (n = 232), plasma cells, CD8^+^ T cells, regulatory T cells (Tregs), M0 macrophages, M2 macrophages, resting mast cells, and eosinophils were significantly upregulated, while activated mast cells and neutrophils were significantly downregulated when compared to those in the low risk subgroup (n = 247) (all P < 0.05; **Figure 6A**).

The expression values for the clinically detectable inflammatory cytokines and chemokines were extracted from GSE65682 and compared between the IRG subgroups. The results showed that *CCL3*, *CSF3* and *IL10* were significantly higher in the high risk subgroup (n = 232), while *CCL5*, *IL15*, *IL1B*, *IL1RN* and *VEGFA* were significantly lower in the high risk subgroup (n = 232) (all P < 0.05; **Figure 6B**). *TNF* exhibited a trend toward a lower expression in the high risk subgroup, but the difference was not significant (P = 0.08; **Figure 6B**). Elevated ratios of anti-inflammatory to pro-inflammatory cytokines (e.g. IL10/TNF) are proposed markers of sepsis-induced immunosuppression, and are associated with multiple organ failure and increasing sepsis severity and mortality (20, 21). In our study, we found that the IRG signature was positively correlated with *IL10*/*TNF* ratio (r = 0.23, P = 6.8e-7; **Figure 6C**).

### Assessment of biological pathways associated with the IRG signature

**Figure 7:**
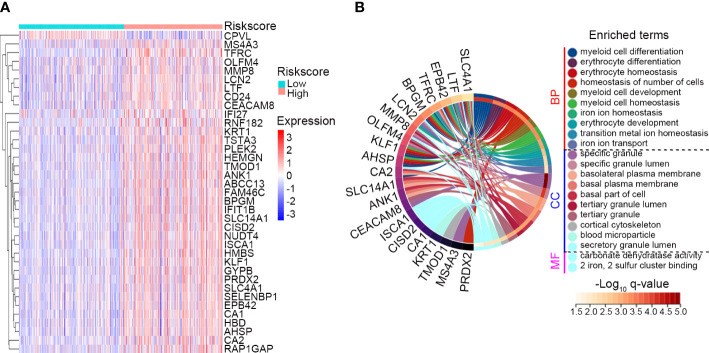
Assessment of biological pathways associated with the IRG signature. **(A)** The expression profile of the DEGs between the low risk (n = 247) and high risk (n = 232) subgroups classified by the IRG signature in GSE65682. The screening criteria were set as adj. P-value < 0.05 and |Log_2_ FC| ≥ 1. **(B)** GO analysis of the identified DEGs. Top 10 significant biological process (BP), top 10 significant cellular component (CC) and 2 significant molecular function (MF) of GO analysis were shown.

DEGs between the IRG subgroups in GSE65682 were identified, and a total of 36 genes were significantly upregulated and 1 gene was significantly downregulated in the high risk subgroup (**Figure 7A**). These genes were subjected to GO analysis to explore biological pathways associated with the IRG signature. Genes enriched for biological process were mainly associated with myeloid cell differentiation, development and homeostasis, erythrocyte differentiation and homeostasis, and iron ion transport and homeostasis (**Figure 7B**). Genes enriched for cellular component were mainly involved in specific/secretory/tertiary granule lumen, basal/basolateral plasma membrane, and cortical cytoskeleton (**Figure 7B**). Genes enriched for molecular function were involved in carbonate dehydratase activity and 2 iron, 2 sulfur cluster binding (**Figure 7B**). The complete list of the enriched terms is shown in **Table S7**.

### Validation of IRG signature genes in clinical specimens

**Figure 8:**
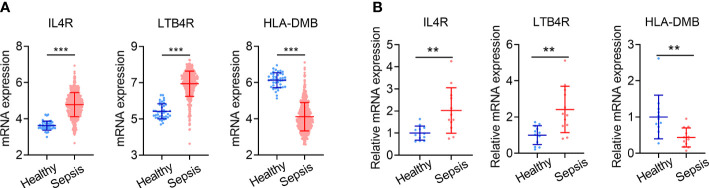
Validation of IRG signature genes in clinical specimens. **(A)** Expression levels of *IL4R*, *LTB4R* and *HLA-DMB* in sepsis patients (n = 479) and healthy controls (n = 42) in GSE65682. **(B)** Expression levels of *IL4R*, *LTB4R* and *HLA-DMB* analyzed by qPCR in 24 cases of clinical specimens (12 sepsis patients and 12 healthy controls). Data is shown as mean ± SD. **P < 0.01, ***P < 0.001.

The expression levels of the three genes comprising the IRG signature in the GSE65682 dataset are shown in **Figure 8A**. Compared with healthy controls (n = 42), *IL4R* and *LTB4R* were significantly upregulated in sepsis patients (n = 479), while *HLA-DMB* was significantly downregulated (all P < 0.001). We validated the expression of these genes in PBMCs from 12 sepsis patients and 12 healthy controls using qPCR. The qPCR data exhibited the same pattern of expression of these genes as the GSE65682 dataset, with *IL4R* and *LTB4R* being expressed at significantly higher levels in sepsis cases and *HLA-DMB* exhibiting the opposite trend (all P < 0.01; **Figure 8B**).

## Discussion

A biomarker that would be capable of predicting sepsis disease outcome would be of considerable clinical importance. There is emerging evidence for genetic associations with susceptibility to and outcomes of infectious diseases, including sepsis (22, 23). Blood transcriptional profiling has led to substantial advances in sepsis treatment (24). Although promising new diagnostic biomarkers have been discovered from the application of blood transcriptomics to sepsis, patient selection for interventional trials and prediction of patient outcomes in sepsis continue to be driven by clinical signs (9, 25, 26). Attributing disease signatures to patient outcomes and establishing the functional significance of any genetic associations have so far been limited in such studies.

In the present study, we identified and validated an IRG signature for 28-day survival prediction in sepsis based on two publicly available datasets (GSE65682 and E-MTAB-4451). By applying the risk score model of the IRG signature to sepsis patients, a clear separation was observed in survival curve between patients in the high risk and low risk subgroups of the training, testing and entire set of GSE65682. The predictive capability of the IRG signature was also externally validated in another sepsis dataset, namely E-MTAB-4451. In GSE65682, the IRG signature was significantly associated with source of infection and Mars endotype, but not age, gender, history of diabetes mellitus, or thrombocytopenia. Mars endotype is a novel molecular classification of sepsis patients based on blood transcriptomics which was carried out within the wide context of the Molecular Diagnosis and Risk Stratification of Sepsis (MARS) project, a prospective observational cohort study in the mixed ICUs of two tertiary teaching hospitals (Academic Medical Center in Amsterdam, Netherlands, and University Medical Center in Utrecht, Netherlands) (9, 27, 28). Of note, we performed Cox regression analysis, and the results suggested that the prognostic value of the IRG signature was independent of age, gender and Mars endotype. Finally, it was fascinating to find that the IRG signature had greater predictive power than Mars endotype in the ROC analysis, though the difference was not statistically significant. Moreover, when combined with Mars endotype, the IRG signature showed even better predictive ability. These results indicate that the combination of the identified IRG signature and Mars endotype may help improve survival prediction in sepsis patients.

The IRG signature consists of three individual genes, namely *LTB4R*, *HLA-DMB* and *IL4R*. In GSE65682, the data analysis demonstrated that *IL4R* and *LTB4R* were significantly upregulated in sepsis, while *HLA-DMB* was significantly downregulated compared to healthy controls. We further validated the expression of these genes in 24 cases of clinical specimens using qPCR, and the results of qPCR were in accordance with those of the above bioinformatics analyses. Although some of these genes are reportedly associated with sepsis, their biological roles have not been thoroughly investigated. For example, by analyzing multiple microarray datasets, Huang et al. (29) and Zhou et al. (30) simultaneously identified *LTB4R* as a promising diagnostic biomarker of sepsis. Moreover, *LTB4R* was found to be associated with mortality of sepsis patients (29). In patients with sepsis who exhibited features of immune depression, inverse correlations between *HLA-DR* (including *HLA-DMA* and *HLA-DMB*) and *PDE4D* expression were observed, indicating the role of cAMP-related HLA-DR modulation in sepsis (31). Another candidate, *IL4R*, encoding the alpha chain of the interleukin-4 receptor that can bind IL4 and IL13, has not been previously reported to be associated with infectious diseases. Our findings suggest that they deserve further investigation to clarify their potential as biomarkers in sepsis.

Historically, sepsis was considered to consist of an initial hyper-inflammatory phase followed by an anti-inflammatory or immunosuppressive phase (32). This biphasic paradigm has been challenged by numerous recent reports, and it has now become evident that pro-inflammatory and anti-inflammatory phases can occur during variable time points in the disease course of sepsis (33, 34). Sepsis-related immune cell death and compromised immune cell function were suggested to be evidence of immunosuppression (35). For example, early activation of Tregs during *Staphylococcus aureus* sepsis was found to induce CD4^+^ T cell impairment and increase susceptibility to secondary pneumonia (36). Moreover, macrophages play an important role in regulating the host’s immune balance and inflammatory response in sepsis, and an imbalance between M1-like and M2-like macrophages has been suggested to contribute to the occurrence of sepsis (37). In the present study, we inferred from transcriptomics data the enrichment of Tregs and M2 macrophage in the IRG high risk subgroup, suggesting possible involvement of Tregs and macrophage in sepsis-related immunosuppression as early as 24h amid sepsis occurrence. Various molecules, including cytokines, chemokines, and acute phase reactants are involved in the process of sepsis (38–40). The cytokine and chemokine analysis in our study revealed that anti-inflammatory cytokine (*IL10*) showed higher expression level in the high risk subgroup classified by the IRG signature, while pro-inflammatory cytokines (*IL1B* and *IL15*) were downregulated in the high risk subgroup. Moreover, the ratio of *IL10/TNF*, a reported indicator of sepsis-induced immunosuppression and sepsis-related mortality, was significantly correlated with the IRG risk score. Taken together, we concluded that the IRG signature could mirror the immunological status of sepsis patient, which may aid in stratifying suitable patients for immune-modulating therapy and evaluating the therapeutic efficacy.

We also performed differential analysis to screen DEGs between IRG subgroups in GSE65682. It is interesting to note that several DEGs (namely *OLFM4*, *LCN2*, *MMP8* and *LTF*) identified in the present study were coincidently uncovered by other groups as potential biomarkers capable of distinguishing septic shock from non-septic shock in postsurgical patients (41, 42).The above genes were able to discern between both shock conditions better than other biomarkers used for diagnosis of these conditions, such as procalcitonin, C-reactive protein or neutrophils (41). We further performed GO analysis of the identified DEGs, and the result suggested their possible involvement in myeloid cell differentiation and iron ion homeostasis among others, providing clues regarding the underlying biological mechanisms of the IRG signature.

The limitations should be acknowledged for our study. First, the predictive model was established merely based on transcriptomics data. The AUCs of the IRG signature were 0.704, 0.601, 0.648 and 0.619 for the training, testing, entire (training set plus testing set) sets of GSE65682 and E-MTAB-4451, respectively. There is still a long way to go before utilizing the IRG signature as a clinically useful biomarker, particular given that performance could very likely be lower in a sepsis patient with different disease etiology or genetic background. Other system-based omics data or clinical characteristics of patients should also be included to increase its predictive power when applied it for clinical practice. Second, since changes of the disease state of sepsis are rapid and dynamic, longitudinal studies of the molecular changes and how those changes might impact patient outcomes and response to therapies are required. Third, based on bulk transcriptomics data, CIBERSORT deconvolution algorithm may not accurately identify immune cell subpopulations. It is necessary to use flow cytometry or single-cell RNA-Seq methods to verify our results. Finally, we have limited experimental data and lack information on the regulatory mechanisms and functional roles of the three individual genes of the IRG signature.

## Conclusions

In conclusion, the present study identified an IRG signature capable of predicting the 28-day mortality in sepsis patients. The innovative IRG signature may aid in stratifying risky sepsis patients and evaluating patients’ immune state. Also, clinical investigations in additional sepsis patient cohorts are needed to validate and elaborate its clinical utility.

## Data availability statement

The original datasets included in this study are available in the GEO (http://www.ncbi.nlm.nih.gov/geo/) and ArrayExpress (https://www.ebi.ac.uk/arrayexpress/) databases. Secondary data generated and analyzed during this study are contained in this published article or **Supplementary Materials**, further inquiries can be directed to the corresponding authors.

## Author contributions

YP and HZ: Conceptualization; YP, JZ, QH, and FY: Data curation; YP, QW, and HL: Formal analysis; YP, LW, QC, and HZ: Methodology; FZ, CF, and HZ: Supervision; FZ, CF, and HZ: Writing (review and editing). All authors contributed to the article and approved the submitted version.

## Supplementary material

Supplementary Figure 1 WGCNA of sepsis patients (n = 479) and healthy controls (n = 42) in GSE65682. **(A)** Sample clustering to detect outliers. **(B)** Analysis of the scale-free fit index (left panel) and the mean connectivity (right panel) for various soft-threshold powers. R^2^ = 0.988 and β = 8 (red line) were selected for the subsequent analysis. **(C)** Cluster dendrogram (unmerged) of co-expression network modules ordered by a hierarchical clustering of genes and based on the 1-TOM matrix. Different colors represent different modules. **(D)** Clustering of module eigengenes. Modules whose distance were less than 0.25 (red line) were merged. **(E)** Cluster dendrogram (merged) of co-expression network modules. **(F)** Module membership (MM) versus gene significance (GS) plot of the brown module.

Supplementary Figure 2 ROC analysis of the sensitivity and specificity of 28-day survival prediction in E-MTAB-4451 (n = 106).

Supplementary Figure 3 Immune cell fractions of sepsis patients in GSE65682 (n = 479) analyzed by CIBERSORT method.
