# Supplementary figures and images for: An immune-related gene signature predicts the 28-day mortality in patients with sepsis
Source: Front Immunol. 2023 Mar 23;14:1152117. doi: 10.3389/fimmu.2023.1152117 (PMC10076848; doi:10.3389/fimmu.2023.1152117)

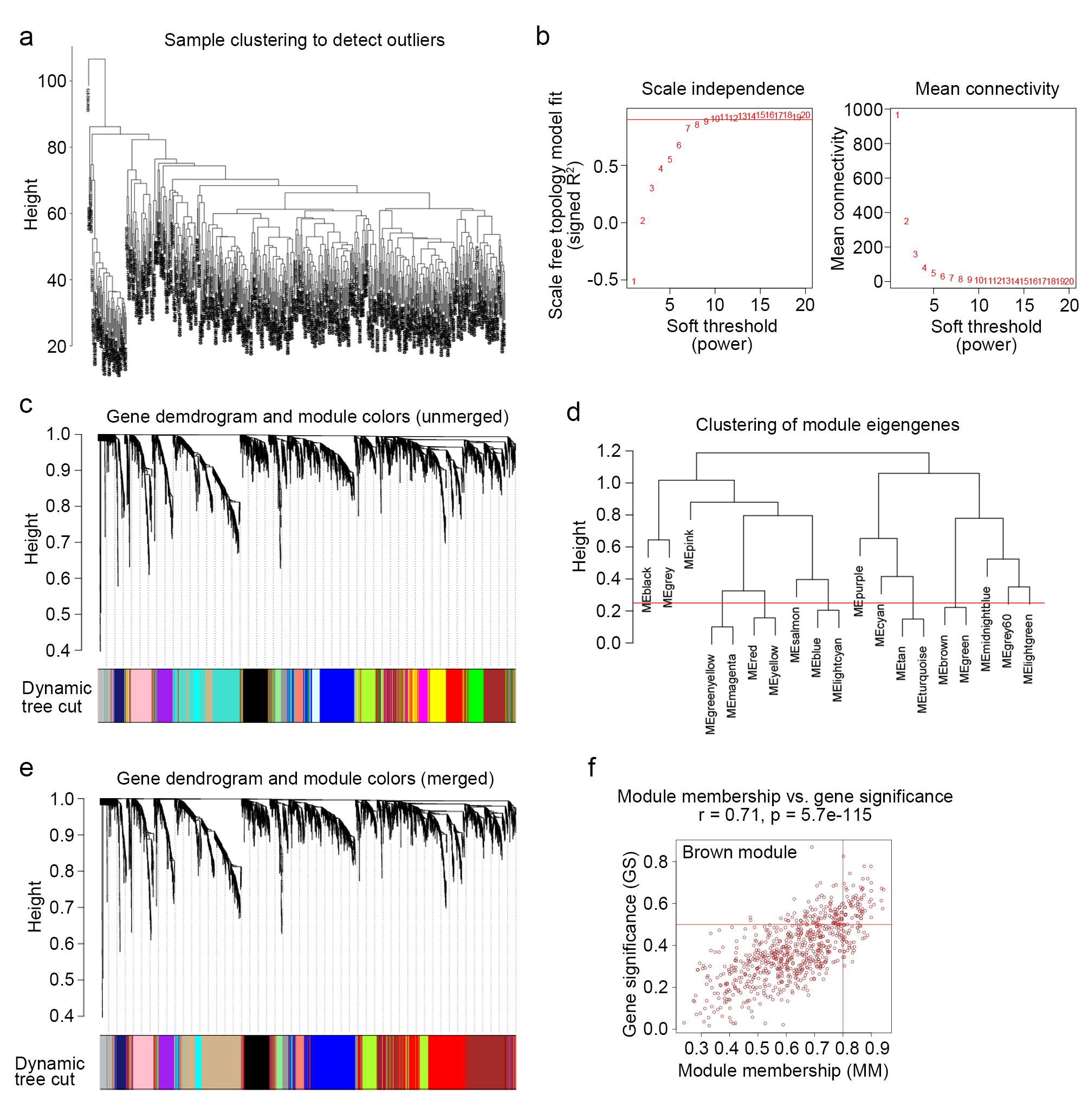

Supplement: Supplementary Figure 1 — WGCNA of sepsis patients (n = 479) and healthy controls (n = 42) in GSE65682. (A) Sample clustering to detect outliers. (B) Analysis of the scale-free fit index (left panel) and the mean connectivity (right panel) for various soft-threshold powers. R2 = 0.988 and β = 8 (red line) were selected for the subsequent analysis. (C) Cluster dendrogram (unmerged) of co-expression network modules ordered by a hierarchical clustering of genes and based on the 1-TOM matrix. Different colors represent different modules. (D) Clustering of module eigengenes. Modules whose distance were less than 0.25 (red line) were merged. (E) Cluster dendrogram (merged) of co-expression network modules. (F) Module membership (MM) versus gene significance (GS) plot of the brown module. [file Image_1.tif]

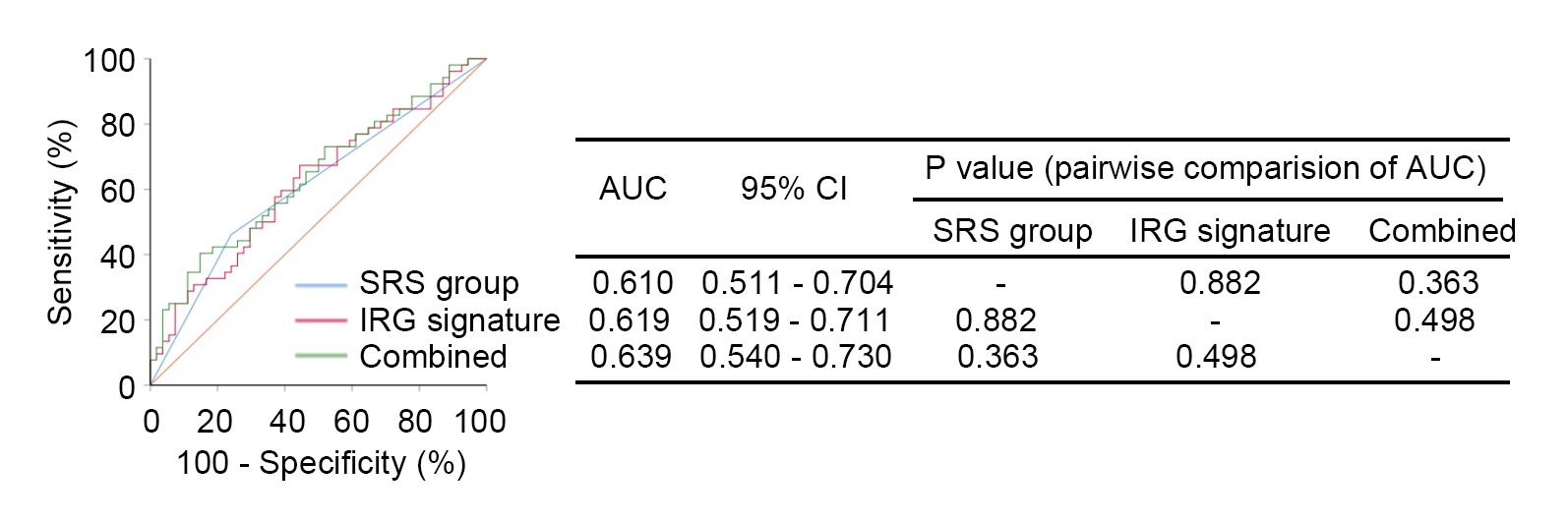

Supplement: Supplementary Figure 2 — ROC analysis of the sensitivity and specificity of 28-day survival prediction in E-MTAB-4451 (n = 106). [file Image_2.tif]

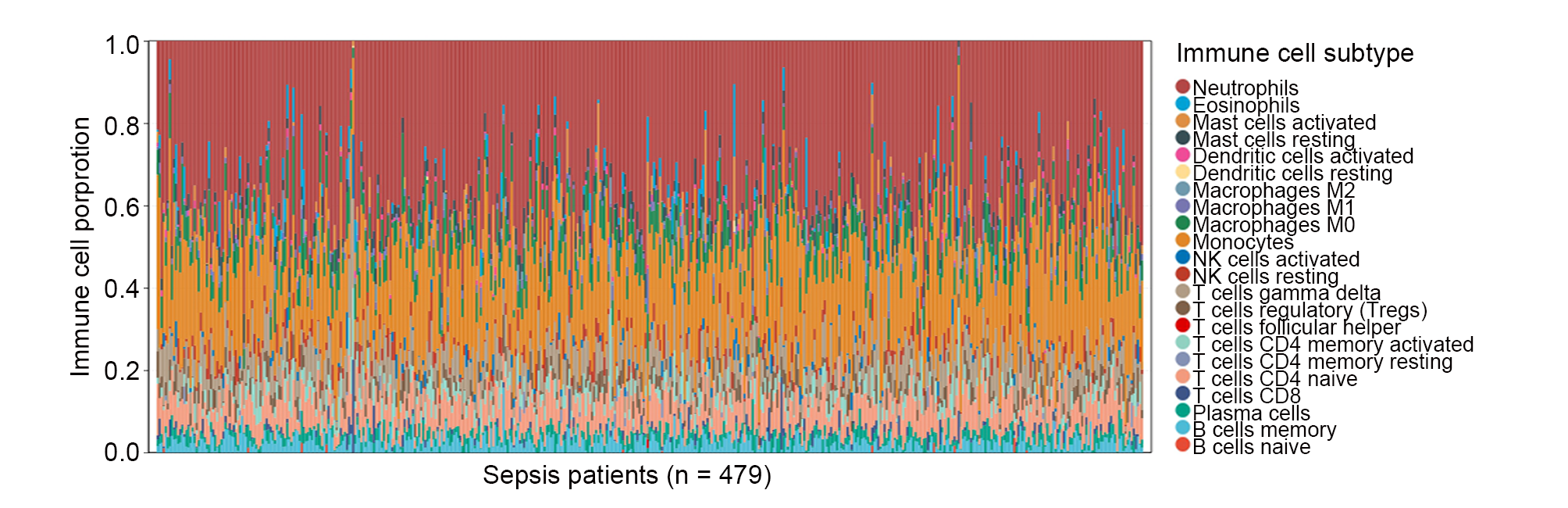

Supplement: Supplementary Figure 3 — Immune cell fractions of sepsis patients in GSE65682 (n = 479) analyzed by CIBERSORT method. [file Image_3.tif]
