# Supplementary material for: An immune-related gene signature predicts the 28-day mortality in patients with sepsis
Source: Front Immunol. 2023 Mar 23;14:1152117. doi: 10.3389/fimmu.2023.1152117 (PMC10076848; doi:10.3389/fimmu.2023.1152117)
Supplement: Supplementary file 5 [file Table_2.docx]

**Table S2.** A set of 27 clinically detectable inflammatory cytokines and chemokines included in this study.

| **Cytokine** | **Gene symbol** |
| --- | --- |
| IL-1beta | *IL1B* |
| IL-1ra | *IL1RN* |
| IL-2 | *IL2* |
| IL-4 | *IL4* |
| IL-5 | *IL5* |
| IL-6 | *IL6* |
| IL-7 | *IL7* |
| IL-8 | *CXCL8* |
| IL-9 | *IL9* |
| IL-10 | *IL10* |
| IL-12(p70) | *IL12A/IL12B* |
| IL-13 | *IL13* |
| IL-15 | *IL15* |
| IL-17 | *IL17A* |
| Eotaxin | *CCL11* |
| Basic FGF | *FGF2* |
| G-CSF | *CSF3* |
| GM-CSF | *CSF2* |
| IFN-gamma | *IFNG* |
| IP-10 | *CXCL10* |
| MCP-1 | *CCL2* |
| MIP-1 alpha | *CCL3* |
| PDGF-BB | *PDGFRB* |
| MIP-1beta | *CCL4* |
| RANTES | *CCL5* |
| TNF-alpha | *TNF* |
| VEGF | *VEGFA* |
