# Supplementary material for: An immune-related gene signature predicts the 28-day mortality in patients with sepsis
Source: Front Immunol. 2023 Mar 23;14:1152117. doi: 10.3389/fimmu.2023.1152117 (PMC10076848; doi:10.3389/fimmu.2023.1152117)
Supplement: Supplementary file 6 [file Table_3.docx]

**Table S3.** Primers used in this study.

| Primers | Sequence (5’-3’) |
| --- | --- |
| IL4R (F) | ACGTGGTCAGTGCGGATAAC |
| IL4R (R) | CTGAAATCTGCCGGGTCGTT |
| LTB4R (F) | CTCTAGGGAAGGGACCATGGAG |
| LTB4R (R) | CTGACAGCAGGATGATAGCCA |
| HLA-DMB (F) | ACTGAACTCCCGGCATCTTT |
| HLA-DMB (R) | AAATTCGCAAGGGGCCATCT |
| GAPDH (F) | CAAGGTCATCCATGACAACTTTG |
| GAPDH (R) | GTCCACCACCCTGTTGCTGTAG |
